# Convention of the Kentucky State Medical Society

**Published:** 1877-06

**Authors:** 


### Convention of the Kentucky State Medical Society.

Dr. Baker, of Shelbyville, offered the following resolutions, which were adopted unaminously:

*Resolved*, That this society is in full accord with the American Medical College convention, seeking to elevate the standard of medical education in this country.

*Resolved*, That summer schools, which enable students to graduate after from eight to nine months' study, are exerting an evil influence upon the profession.

*Resolved*, That a winter and summer course by the same school, and graduation at the end of each, tends to deteriorate the standing of the medical profession.

Dr. Larrabee, after a few remarks in explanation, read the following resolutions, which were adopted:

Whereas, It has come to our knowledge that a bill, known as the "Morrison bill," for the discontinuance of the "tariff on quinine," is at this time before the Committee on Ways and Means, in the Congress of the United States; and whereas, the welfare of a large portion of the people in the Western States and Territories is concerned in the issue of this bill, as well as any movement which will enable them to obtain quinine at a less cost than the enormous prices now paid by the consumer; and whereas, the opposition to this bill set forth by the manufacturers and trade does not represent the desire of those who are engaged in the relief of suffering and want, but ignores entirely the necessities of this large population, many of whom are engaged in cultivating the soil and opening up new resources of wealth to the Government in malarial districts; and whereas, principles of justice and humanity alike demand free quinine and an open market for the competition of European manufacturers; and whereas, we, the members of the Kentucky State Society, in convention assembled, represent the sentiments of the people of this Commonwealth upon this important subject; therefore be it resolved:

*First*—That we indorse the "Morrison bill," and do further pray that your honorable body will hear our petition.

*Second*—That a copy of these resolutions (printed) be sent to similar organizations of physicians' meetings in the various States.

*Third*—That these resolutions, with the signatures affixed, be furnished to our Senators and Representatives in Congress of the United States at its next meeting.

Notice was given of a number of volunteer papers to be read during the day.

